# Prospective study of plasma levels of coenzyme Q10 and lung cancer risk in a low‐income population in the Southeastern United States

**DOI:** 10.1002/cam4.3637

**Published:** 2021-02-06

**Authors:** Chris Shidal, Hyung‐Suk Yoon, Wei Zheng, Jie Wu, Adrian A. Franke, William J. Blot, Xiao‐Ou Shu, Qiuyin Cai

**Affiliations:** ^1^ Division of Epidemiology Department of Medicine Vanderbilt Epidemiology Center Vanderbilt‐Ingram Cancer Center Vanderbilt University School of Medicine Nashville TN USA; ^2^ University of Hawaii Cancer Center Honolulu HI USA

**Keywords:** biomarkers, coenzyme Q10, epidemiology, lung cancer

## Abstract

**Background:**

Coenzyme Q10 (CoQ10) is a ubiquitous molecule in living organisms serving as a cofactor in energy production. Epidemiological studies have reported low CoQ10 levels being associated with an increased risk of various cancers. We conducted the first study to evaluate the association of CoQ10 concentrations with lung cancer risk.

**Methods:**

A nested case‐control study including 201 lung cancer cases and 395 matched controls from the Southern Community Cohort Study was conducted. Plasma CoQ10 levels were measured using high‐performance liquid chromatography with photo‐diode array detection. Conditional logistic regression models were applied to estimate odds ratios (ORs) and 95% confidence intervals (CIs) for the association between plasma CoQ10 levels and lung cancer risk.

**Results:**

Plasma CoQ10 concentration was inversely associated with the risk of lung cancer. After adjusting for age, sex, race, and socioeconomic status, the OR (95% CI) comparing the third to first tertile was 0.57 (0.36–0.91, *P* for trend = 0.02). Further adjustments for smoking, alcohol, chronic obstructive pulmonary disease, and body mass index attenuated the point estimate slightly (OR = 0.60, 95% CI = 0.34–1.08, *P* for trend = 0.11), comparing third to first tertiles. Stratified analyses identified a significant inverse association between plasma CoQ10 levels and lung cancer risk in current smokers, but not in former/never smokers. The association was more evident in cases who were diagnosed within 1 year of blood draw than in cases diagnosed after 1 year.

**Conclusions:**

Low plasma CoQ10 was significantly associated with increased lung cancer risk, particularly among current smokers. The stronger association seen shortly following the blood draw suggests that CoQ10 may be related to disease progression.

## Graphical abstract

We conducted a nested case‐control study to determine the association between plasma coenzyme Q10 and lung cancer risk. Low coenzyme Q10 was associated with a greater risk of lung cancer among current smokers and participants diagnosed within 1 year following blood draw.

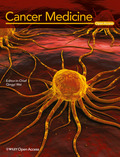

## 1 INTRODUCTION

Lung cancer caused nearly 150,000 deaths in the United States in 2019 and is the leading cause of cancer‐related mortality, accounting for a higher number of deaths than colon, breast, and prostate cancers combined.^1^ While several risk factors for lung cancer have been well characterized (i.e., smoking), there is a need to better understand alternative risk factors, particularly with regard to how they interact with known risk factors as well as the underlying mechanisms of carcinogenesis.

Coenzyme Q10 (CoQ10) is found throughout the body in both its oxidized (ubiquinone) and reduced (ubiquinol) forms, where it functions in ATP biosynthesis.^2^ CoQ10 is synthesized de novo in virtually all cells and is necessary for energy‐requiring functions including proliferation, apoptosis, immune activity, and cell mobility.^3^, ^4^, ^5^ The ubiquitous nature of CoQ10 and its explicit involvement with energy production, inflammation,^6^ and antioxidant activity^7^ present several unique roles that CoQ10 may play in cancer pathogenesis and progression.

Epidemiological evidence has found inconsistent associations between CoQ10 levels and cancer risk. In a Chinese population, Cooney et al. reported an inverse association between plasma CoQ10 levels and breast cancer risk.^8^ Conversely, a study within the Multiethnic Cohort Study (MEC) found a positive association between CoQ10 and breast cancer risk in postmenopausal women.^9^ Additional studies have revealed inconsistencies in the associations between CoQ10 and carcinogenesis, which seem to be dependent upon several key factors including tissue type, the study population, and the methods for risk assessment. For example, a report from the MEC described no significant associations between plasma CoQ10 levels and prostate cancer risk;^10^ yet, a hospital‐based case‐control study in melanoma reported that plasma CoQ10 was lower in melanoma cases than controls, and that lower plasma CoQ10 was associated with disease progression.^11^ Studies have observed that CoQ10 concentrations decrease with age^12^ and vary by geographical location,^13^ suggesting dietary or other environmental factors may influence systemic CoQ10. No study has been conducted to evaluate the association of circulating CoQ10 levels with lung cancer risk. Thus, we conducted a nested case‐control study within the Southern Community Cohort Study (SCCS), a low‐income population living in the Southeastern United States, to assess the association between plasma CoQ10 levels and lung cancer risk.

## 2 MATERIALS AND METHODS

### 2.1 Study population and data collection

The SCCS is a prospective cohort study consisting of approximately 85,000 adult men and women living in the southern United States, between the ages of 40 and 79. A detailed description regarding the full study design appears elsewhere.^14^ Briefly, participants were recruited from 12 southeastern American states including Alabama, Arkansas, Florida, Georgia, Kentucky, Louisiana, Mississippi, North Carolina, South Carolina, Tennessee, Virginia, and West Virginia. Participants were recruited from both Community Health Centers (CHC, 86%) serving low‐income and insured households and through written materials by mail (14%) within the defined geographical areas. Baseline data were collected through computer‐assisted in‐person interviews, and the cohort was followed for cancer occurrence and incidence of mortality through record linkage to the state cancer registries and/or National Death Index databases. This nested case‐control study included 201 incident lung cancer cases who were diagnosed with lung cancer (the International Statistical Classification of Disease (ICD) codes for lung cancer, ICD‐10: C340‐C349) between 2002 and 2009 and provided a blood sample to the study at baseline enrollment. Controls (*N* = 395) were randomly selected from cancer‐free SCCS participants and individually matched to cases at a 2:1 ratio on age (±2 years), race (African American or European American), and sex, as well as date (±6 months) and site (CHC) of study enrollment. The Institutional Review Boards of Vanderbilt University and Meharry Medical College approved the study's protocol, and written consent was obtained from all study participants.

### 2.2 Laboratory assays

Biological specimens were provided by study participants during enrollment at CHC. Participants were asked to donate a total of 20 ml of blood from which plasma samples were isolated and stored at −80°C until biomarker analyses. Plasma samples were extracted with hexane, and extracts were then stored at −80°C prior to analysis of the specimen by high‐performance liquid chromatography (HPLC) with photo‐diode array detection, as previously described.^8^ Briefly, total CoQ10 was determined by HPLC (Model Spectra, Thermo Fisher) with pre‐column electrochemical oxidation and post‐column UV detection (275 nm). δ‐tocopheryl laurate was used as an internal standard to adjust the final CoQ10 concentrations for each sample. The range of interassay variability was 5–7%. Detection and quantitation by HPLC were performed in a blinded sampling method as to reduce experimental bias. The concentrations of CoQ10 present in the plasma of study participants were assessed and are provided as ng/ml.

### 2.3 Statistical analysis

Our study population included a total of 596 subjects, including 201 incident lung cancer cases and 395 control subjects; each case was matched with up to two controls as described previously. Comparative sample collection dates ensured similar specimen storage duration. Demographics, including risk factors, socioeconomic status, and CoQ10 concentrations, were compared between controls and cases, and *p* values were derived using Student's *t*‐test for continuous variables or Chi‐square test for categorical variables. Conditional logistic regression models were used to estimate odds ratios (ORs) and corresponding 95% confidence intervals (95% CIs) between plasma CoQ10 and the risk of lung cancer after adjusting for potential confounders including age at baseline, race (European American and African American), sex (male and female), smoking status (current, former, and never), pack‐years (<30 years, ≥30 years), alcohol consumption (heavy, moderate, and nondrinker), education (<12 years, completed high school, vocational/technical school, university degree, or higher), household income (<$15,000 per year, $15,000–$24,999 per year, and ≥$25,000 per year), diagnosis of chronic obstructive pulmonary disease (COPD) (yes and no), and body mass index (BMI) (<25 kg/m^2^, 25–30 kg/m^2^, and >30 kg/m^2^). Plasma CoQ10 levels were categorized into tertiles based on race‐ and sex‐specific distributions among controls; the lowest tertile was chosen as the reference. Stratified analyses were conducted by gender, race, smoking status, and time between blood collection to diagnosis. Cut‐off values for statistical significance was set at *p* < 0.05 for all statistical analyses. Statistical analyses were conducted using SAS software (SAS Institute, Cary, NC).

## 3 RESULTS

The demographics of our study population are presented in Table 1. Our study population represents a cohort with low socioeconomic status, as evidenced by the high percentage of study participants with a low household income (<$15,000 per year), as well as a high percentage of participants with <12 years of schooling. The proportion of current smokers was significantly higher in cases when compared with their matched controls (72.1% vs. 41.8%). Pack‐years was also significantly higher in cases than in matched controls (29.0 pack‐years vs. 19.5 pack‐years). Lung cancer cases were more likely to be less educated, earn less income, be alcohol drinkers, have lower BMIs, and a history of COPD compared with controls (Table 1). Median plasma CoQ10 concentrations were significantly lower in cases (973 ng/ml) than controls (1,076 ng/mL) (*p* < 0.01).

**TABLE 1:** Baseline Characteristics of lung cancer cases and controls in the SCCS

	Cases	Controls	*p*‐value*
	(N = 201)	(N = 395)	
Age (mean±SD)	56.8** ± **8.2	56.4** ± **8.2	0.63
Race (N (%))			
African Americans	143 (71.1)	281 (71.1)	—
European Americans	58 (28.9)	114 (28.9)	
Gender (N (%))			
Men	118 (58.7)	230 (58.2)	0.91
Women	83 (41.3)	165 (41.8)	
Smoking status (N (%))			
Current	145 (72.1)	165 (41.8)	<0.01
Former	43 (21.4)	120 (30.4)	
Never	10 (5.0)	105 (26.6)	
Pack‐years^a^ (median (IQR))	29.0 (16.2–49.5)	19.5 (9.3–37.0)	<0.01
Alcohol consumption (N (%))			
Heavy	49 (24.4)	62 (15.7)	0.03
Moderate^b^	57 (28.4)	138 (34.9)	
Nondrinker	90 (44.8)	190 (48.1)	
Education (N (%))			
Less than 12 years	102 (50.7)	176 (44.6)	0.17
Completed high school	59 (29.4)	119 (30.1)	
Vocational/technical school	32 (15.9)	67 (17.0)	
University degree or higher	5 (2.5)	28 (7.1)	
Household income (N (%))			
<$15,000	143 (71.1)	266 (67.3)	0.78
$15,000–$24,999	40 (19.9)	85 (21.5)	
≥$25,000	14 (7.0)	35 (8.9)	
BMI (kg/m^2^)			
<25	98 (48.8)	112 (28.4)	<0.01
25–30	56 (27.9)	114 (28.9)	
≥30	41 (20.4)	161 (40.8)	
COPD^c^ (N (%))			
No	162 (80.6)	357 (90.4)	<0.01
Yes	36 (17.9)	33 (8.4)	
Plasma CoQ10 (ng/ml, median (IQR^d^))	973 (712–1,266)	1076 (798–1,353)	<0.01

^a^ Included former/current smoker.

^b^ Moderate alcohol intake is defined as >0 but ≤2 drink/day for men or ≤1 drink/day for women.

^c^ Ever diagnosed with emphysema or chronic bronchitis.

^d^Interquartile range.

* An analysis of co‐variance was used to investigate differences between case and control by the *t*‐test procedure for continuous variables and Chi‐square test for categorical variables.

Table 2 compared median plasma CoQ10 concentrations between cases and controls on several demographic and lifestyle factors. Significant differences in plasma CoQ10 levels were observed between cases and controls among young (≤56 years, *p* = 0.03), European American (*p* = 0.01), women (*p* = 0.03), currently smoking (*p* = 0.03), moderate alcohol consumption (*p* < 0.01), lower level of education (vocational/technical or university degree, *p* = 0.03 and *p* = 0.05, respectively), household income between $15,000 and 24,999 (*p* = 0.05), and BMI between 25 and 30 kg/m^2^ (*p* = 0.04) subgroups. No significant differences in plasma CoQ10 concentration were observed between cases and controls in subgroup analyses stratified by pack‐years or history of COPD (Table 2). We also evaluated whether these demographic and lifestyle factors affected plasma CoQ10 levels among controls (Table 2). Plasma CoQ10 concentration was higher in African Americans than in European Americans (*p* = 0.01). Alcohol consumption was also positively associated with higher plasma CoQ10 (*p* < 0.01). We did not observe significant differences in subgroup analyses among controls by age, gender, socioeconomic factors, smoking status, BMI, or a history of COPD (Table 2). Interestingly, we did observe a significant difference when age was stratified by ≤60 years and >60 years (1124 ng/ml and 1005 ng/ml, respectively, *p* < 0.01).

**TABLE 2:** Median plasma CoQ10 level (ng/ml) between cases and controls by covariates in the SCCS

	Cases	Controls	*p*‐value*	*p*‐value**
	Median (ng/ml)	Median (ng/ml)		
Age				
≤56	946	1114	0.03	0.16
>56	1001	1023	0.13	
Race				
African Americans	1035	1097	0.15	0.01
European Americans	789	980	0.01	
Gender				
Men	1001	1103	0.11	0.14
Women	906	1009	0.03	
Smoking status				
Current	969	1124	0.03	0.36
Former	1052	1032	0.69	
Never	793	1046	0.08	
Pack‐years^a^				
≥30	905	1061	0.37	0.25
<30	1014	1080	0.08	
Alcohol consumption				
Heavy	1103	1253	0.17	<0.01
Moderate^b^	944	1100	<0.01	
Nondrinker	896	996	0.23	
Education				
Less than 11 years	1064	1011	0.78	0.24
Completed high school	919	1075	0.07	
Vocational/technical school	851	1089	0.03	
University degree or higher	603	1207	0.05	
Household income				
<$15,000	1001	1043	0.13	0.60
$15,000–$24,999	821	1163	0.05	
≥$25,000	1030	1070	0.21	
BMI (kg/m^2^)				
<25	971	1076	0.45	0.28
25–30	946	1098	0.04	
≥30	1028	1024	0.13	
COPD^c^				
No	1002	1075	0.08	0.96
Yes	789	1076	0.06	

^a^ Included former/current smoker.

^b^ Moderate alcohol intake is defined as >0 but ≤2 drink/day for men or ≤1 drink/day for women.

^c^ Ever diagnosed with emphysema or chronic bronchitis.

* *p*‐values used to indicate differences between case and control.

** *p*‐values used to indicate differences between subgroups among controls only.

Using conditional logistic regression, we identified a significant inverse association between plasma CoQ10 levels and lung cancer risk (Table 3). We performed regression modeling by constructing three different models, each including additional adjustments from the previous model. First, we conducted conditional logistic regression adjusting for demographic factors, including age, race, gender, and socioeconomic status (income and education). In our second model, we additionally adjusted for smoking status and pack‐years. Finally, we further adjusted for alcohol consumption, history of COPD, and BMI. When adjusting only for demographic factors, we observed an inverse association of plasma CoQ10 levels and lung cancer risk with ORs (95% CIs) of 0.73 (0.48–1.12) and 0.57 (0.36–0.91) for the second and third tertiles, respectively, when compared with the first tertile (*p* for trend = 0.02). Additional adjustment for smoking‐related factors slightly attenuated this association, with ORs (95% CIs) of 0.85 (0.53–1.36) and 0.59 (0.34–1.01) for second and third tertiles, respectively, compared with the first tertile (*p* for trend = 0.06). When we performed adjustments for alcohol, COPD, and BMI, the ORs (95% CI) were 1.01 (0.60–1.68) and 0.60 (0.34–1.08), respectively, for second and third tertiles compared with the first tertile (*p* for trend = 0.11). For increased stringency in our study, we will report only ORs and 95% CIs derived from the final regression model, which adjusts for demographics, smoking, COPD, alcohol, and BMI (i.e., our most adjusted model).

**TABLE 3:** Overall association between plasma CoQ10 level^a^ and lung cancer risk in the SCCS

Plasma CoQ10 level (tertiles)	25–75 percentile^b^	Cases (*N* = 201)	Controls (*N* = 395)	OR (95% CI)^c^	OR (95% CI)^d^	OR (95% CI)^e^
T1	567–802	85	129	1.00 (Ref.)	1.00 (Ref.)	1.00 (Ref.)
T2	975–1140	66	133	0.73 (0.48–1.12)	0.85 (0.53–1.36)	1.01 (0.60–1.68)
T3	1,357–1676	50	133	0.57 (0.36–0.91)	0.59 (0.34–1.01)	0.60 (0.34–1.08)
*p* trend				0.02	0.06	0.11
1 SD increase				0.81 (0.65–0.99)	0.85 (0.67–1.07)	0.87 (0.68–1.11)

Note:

Analysis using conditional logistic regression model.

^a^ Based on the race‐ and sex‐specific tertiles among controls.

^b^ Level 25–75 percentile of each CoQ10’s tertile.

^c^ Adjustment for age, sex, race, income, and education.

^d^ Adjustment for age, sex, race, income, education, smoking status, and pack‐years.

^e^ Adjustment for age, sex, race, income, education, smoking status, pack‐years, alcohol consumption, history of COPD, and BMI.

We performed stratified analyses on subgroups based on race, sex, and smoking status (Table 4). The association between plasma CoQ10 with lung cancer risk was similar between African Americans and European Americans, and between men and women. Comparing third to first tertiles, the ORs (95% CIs) were 0.62 (0.31–1.24) for African Americans and 0.36 (0.08–1.56) for European Americans, and 0.51 (0.24–1.10) for men and 0.59 (0.21–1.67) for women. However, the association between CoQ10 and lung cancer risk was not statistically significant in stratified analyses based on race or sex. We next compared the effect of plasma CoQ10 concentrations on lung cancer risk by smoking status (Table 4). A significant inverse association was observed in current smokers, with an OR of 0.47 (95% CI: 0.26–0.87) for the third tertile compared with the first tertile (*p* for trend = 0.02). We did not observe a significant association in former/never smokers. A significant interaction was observed in our subgroup analysis by smoking status (*p* < 0.01). We also performed stratified analyses based on histological subtype (adenocarcinoma, squamous cell, and small cell lung cancer); the associations were similar between lung cancer subtypes but were not statistically significant (data not shown).

**TABLE 4:** Association between plasma CoQ10 levels^a^ and lung cancer risk by characteristics

Plasma CoQ10 level (tertiles)	African Americans				European Americans			
	25–75 percentile^b^	Cases	Controls	OR (95% CI)^c^	25–75 percentile^b^	Cases	Controls	OR (95% CI)^c^
		(*N* = 143)	(*N* = 281)			(*N* = 58)	(*N* = 114)	
T1	615–831	57	92	1.00 (Ref.)	498–691	28	37	1.00 (Ref.)
T2	1012–1173	47	95	1.01 (0.55–1.85)	873–1054	19	38	0.83 (0.23–3.04)
T3	1378–1727	39	94	0.62 (0.31–1.24)	1266–1543	11	39	0.36 (0.08–1.56)
*p* trend				0.20				0.19
1 SD increase				0.90 (0.67–1.22)				0.73 (0.41–1.30)
*p* for interaction								**<0.01**

Plasma CoQ10 level (tertiles)	Men				Women			
	25–75 percentile^b^	Cases	Controls	OR (95% CI)^c^	25–75 percentile^b^	Cases	Controls	OR (95% CI)^c^
		(*N* = 118)	(*N* = 230)			(*N* = 83)	(*N* = 165)	
T1	599–822	49	75	1.00 (Ref.)	560–748	36	54	1.00 (Ref.)
T2	1010–1187	38	78	0.82 (0.41–1.60)	923–1096	28	55	1.43 (0.54–3.75)
T3	1382–1697	31	77	0.51 (0.24–1.10)	1280–1661	19	56	0.59 (0.21–1.67)
*p* trend				0.09				0.41
1 SD increase				0.84 (0.61–1.17)				0.84 (0.61–1.17)
*p* for interaction								0.72

Plasma CoQ10 level (tertiles)	Current				Former / Never			
	25–75 percentile^b^	Cases	Controls	OR (95% CI)^d^	25–75 percentile^b^	Cases	Controls	OR (95% CI)^d^
		(*N* = 145)	(*N* = 165)			(*N* = 53)	(*N* = 225)	
T1	546–779	62	54	1.00 (Ref.)	603–804	23	74	1.00 (Ref.)
T2	987–1173	49	50	1.02 (0.57–1.82)	970–1,121	15	83	0.51 (0.21–1.21)
T3	1364–1713	34	61	0.47 (0.26–0.87)	1,315–1,673	15	68	0.84 (0.35–2.03)
*p* trend				0.02				0.57
1 SD increase				0.77 (0.59–1.00)				0.91 (0.62–1.33)
*p* for interaction								**<0.01**

^a^ Based on the race‐ and sex‐specific tertiles among controls.

^b^ Level 25–75 percentile of each CoQ10’s tertile.

^c^ Adjustment for age, smoking status, pack‐years, alcohol consumption, education, household income, history of COPD, and BMI.

^d^ Adjustment for age, sex, race, pack‐years, alcohol consumption, education, household income, history of COPD, and BMI.

Finally, we performed stratified analyses on time to diagnosis from the time of blood draw (Table 5). A significant association between plasma CoQ10 levels and lung cancer risk was observed among individuals diagnosed within 1 year, but not in individuals diagnosed within 2 to 3 years or greater than 3 years following blood draw. Compared with the first tertile, the ORs (95% CIs) for the third tertile were 0.17 (0.04–0.73, *p* for trend = 0.02), 0.61 (0.22–1.71, *p* for trend = 0.38), and 0.78 (0.26–2.37, *p* for trend = 0.69), respectively, for diagnosis within 1 year, 2 to 3 years, and longer than 3 years after blood draw. When analyses were conducted separately for participants diagnosed within 2 years or after 2 years following blood draw, the association of plasma CoQ10 concentration with lung cancer risk was not significant in either group (data not shown).

**TABLE 5:** Association between plasma CoQ10 levels^a^ and lung cancer risk by time between blood collection and lung cancer diagnosis

Plasma CoQ10 level (tertiles)	≤1 year follow‐up			
	25–75 percentile^b^	Cases	Controls	OR (95% CI)^c^
		(*N* = 55)	(*N* = 108)	
T1	574–741	24	30	1.00 (Ref.)
T2	953–1154	20	40	0.69 (0.27–1.81)
T3	1364–1661	11	38	0.17 (0.04–0.73)
*p* trend				0.02
1 SD increase				0.46 (0.23–0.90)

	2–3 years follow‐up			
	25–75 percentile^b^	Cases	Controls	OR (95% CI)^c^
		(*N* = 85)	(*N* = 169)	
T1	574–809	38	60	1.00 (Ref.)
T2	997−1140	26	50	0.95 (0.36–2.48)
T3	1319 −1756	21	59	0.61 (0.22–1.71)
*p* trend				0.38
1 SD increase				0.85 (0.58–1.26)

	>3 years follow‐up			
	25–75 percentile^b^	Cases	Controls	OR (95% CI)^c^
		(*N* = 61)	(*N* = 118)	
T1	560–795	23	39	1.00 (Ref.)
T2	972–1143	20	43	1.02 (0.38–2.77)
T3	1378−1673	18	36	0.78 (0.26–2.37)
*p* trend				0.69
1 SD increase				1.17 (0.71–1.92)

^a^ Based on the race‐ and sex‐specific tertiles among controls.

^b^ Level 25–75 percentile of each CoQ10’s tertile.

^c^ Adjustment for age, sex, race, smoking status, pack‐years, alcohol consumption, education, household income, history of COPD, and BMI.

## 4 DISCUSSION

In this first prospective case‐control study nested within the SCCS, we found an inverse association between plasma CoQ10 and lung cancer risk, particularly in cases diagnosed within 1 year following blood collection. This association was also more apparent in individuals currently smoking. We did not observe consistently significant associations between plasma CoQ10 levels and lung cancer risk in our stratified analyses by race or sex.

Several studies have reported that low circulating CoQ10 levels were associated with higher risk of various types of cancer.^8^, ^15^, ^16^ Low CoQ10 concentrations have been clearly observed in various cancer tissues.^11^, ^16^, ^17^, ^18^ CoQ10 supplementation has been reported to reduce cardiotoxicity and improve survival in cancer patients.^19^, ^20^, ^21^ The mechanisms underlying the beneficial effects of CoQ10 supplementation are ambiguous; however, research has suggested that CoQ10 has several anticancer properties including immune‐related effects,^22^, ^23^, ^24^ changes in gene expression,^25^, ^26^ microRNA expression,^27^ and antioxidant activity.^28^, ^29^ Tissues exposed to high levels of oxidative stress (i.e., kidneys) may be at greater risk from CoQ10 deficiency, which would support the notion that oxidative stress can drive carcinogenesis. CoQ10 distribution and redox status are variable and tissue‐dependent; the kidneys contain approximately 66.5 nmol/g with approximately 70% of total CoQ10 in its reduced form, while the lungs contain 9.2 nmol/g with 25% of total CoQ10 in its reduced form.^30^, ^31^ These differences between tissue‐level concentrations may underpin the disparities in epidemiological literature on CoQ10. Further, we found in our study that plasma CoQ10 concentrations among controls were significantly higher in African Americans when compared with European Americans and increased with alcohol consumption. These results highlight potential disparities in plasma CoQ10 among specific subpopulations or based on lifestyle factors.

We observed a significant inverse association between plasma CoQ10 levels and lung cancer risk in current smokers. Cigarette smoke alters the redox status of lung tissues resulting in oxidative DNA damage leading to increased risk of carcinogenesis.^32^, ^33^ While short‐term smoking can be detrimental to predisposed individuals, several studies have provided evidence that redox status^34^ as well as gene expression changes^35^ induced by short‐term cigarette exposures are, in part, reversible. Elevated CoQ10 may provide an antioxidant reservoir capable of scavenging reactive oxygen species (ROS) generated by cigarette smoke in lung tissues. The significance of this association was not observed in former and never smokers, which may indicate that these groups are less reliant on the ROS scavenging activity of CoQ10. Alternatively, several mechanisms exist for detoxifying ROS including enzymatic (e.g., superoxide dismutase) and nonenzymatic (e.g., ascorbic acid) detoxification in which CoQ10 may only play a small role in reducing oxidative stress.

We observed a robust association between plasma CoQ10 levels and lung cancer risk in individuals diagnosed within 1 year after blood collection. While these data may result from a change in dietary intake or environmental factors due to the symptoms of a clinically undiagnosed lung cancer (reverse causation), a true association demonstrating that low plasma CoQ10 may serve as a biomarker for lung cancer risk cannot be excluded. Larger studies would be necessary to provide more definitive evidence supporting the utility of coenzyme CoQ10 as a viable biomarker for lung cancer. Additionally, we tested how sensitive our model was to the time to diagnosis by changing the time period from within 1 year following blood draw to the first 2 years. The association between CoQ10 concentrations and lung cancer risk was not statistically significant using this approach.

The strengths of the current study include the prospective study design in which biological samples were collected before cancer diagnosis, and participants were followed over years to ascertain incident lung cancer occurrence. The comprehensive covariate information available allowed an in‐depth assessment of the confounding and effect modifications of these variables. The inclusion and common performance of several regression models adjusting for demographics, smoking, and additional confounders demonstrate the likelihood of a true association between CoQ10 and lung cancer risk. The SCCS includes populations which are at higher risk for lung cancer when compared to the general population, and further, are an underserved community in the context of healthcare coverage and treatments. Studies utilizing high‐risk and underserved populations present unique opportunities to enrich not only our understanding of processes driving carcinogenesis, but to also benefit the communities themselves.

One limitation of our study is that using plasma concentrations of CoQ10 may not accurately reflect the true concentration and redox status of CoQ10 levels in lung tissues. Additionally, only total CoQ10 was measured; the percentages of oxidized and reduced CoQ10 were not directly observed. Our sample size was large enough to detect significant differences in some stratified analyses, but it is inadequate for detecting moderate interactions. A study utilizing a larger and more diverse cohort may provide additional insights into the association between circulating CoQ10 levels and lung cancer risk.

In conclusion, results from our study showed an inverse association between plasma CoQ10 levels and lung cancer risk, particularly during the year immediately following blood collection. This association was stronger in current smokers when compared to former/never smokers. These data may provide initial evidence of the utility of circulating CoQ10 level as a biomarker for lung cancer. Further studies with a larger sample size are warranted to confirm our findings.

## CONFLICT OF INTEREST

The authors declare no conflict of interest.

## AUTHOR CONTRIBUTIONS

Chris Shidal: Formal analysis, writing‐original draft, writing‐review, and editing. Hyung‐Suk Yoon: Formal analysis, writing‐review, and editing. Wei Zheng: Conceptualization, resources, data curation, funding acquisition, writing‐review, and editing. Jie Wu: Data curation, writing‐review, and editing. Adrian Franke: Data curation, writing‐review, and editing. William Blot: Conceptualization, resources, data curation, funding acquisition, writing‐review, and editing. Xiao‐Ou Shu: Funding acquisition, writing‐review and editing. Qiuyin Cai: Conceptualization, resources, data curation, supervision, writing‐original draft, writing‐review, and editing.

## FINANCIAL SUPPORT

Work was supported in part by NIH grants T32CA160056 (XOS), U01CA202979 (WB and WZ), P30CA71789 (AF), and P30CA068485 (QC).

## PRECIS

We conducted a nested case‐control study to determine the association between plasma coenzyme Q10 level and lung cancer risk. Low coenzyme Q10 was associated with a greater risk of lung cancer among current smokers and participants diagnosed within 1 year following blood draw.

## Data Availability

Data used in the present study can be requested through the SCCS Online request System (https://ors.southerncommunitystudy.org).
